# PHLPP regulates hexokinase 2-dependent glucose metabolism in colon cancer cells

**DOI:** 10.1038/cddiscovery.2016.103

**Published:** 2017-01-23

**Authors:** Xiaopeng Xiong, Yang-An Wen, Mihail I Mitov, Mary C Oaks, Shigeki Miyamoto, Tianyan Gao

**Affiliations:** 1 Markey Cancer Center, University of Kentucky, Lexington, KY, USA; 2 Department of Biology, University of Kentucky, Lexington, KY, USA; 3 Department of Pharmacology, University of California, San Diego, La Jolla, CA, USA; 4 Department of Molecular and Cellular Biochemistry, University of Kentucky, Lexington, KY, USA

## Abstract

Increased glucose metabolism is considered as one of the most important metabolic alterations adapted by cancer cells in order to generate energy as well as high levels of glycolytic intermediates to support rapid proliferation. PH domain leucine-rich repeat protein phosphatase (PHLPP) belongs to a novel family of Ser/Thr protein phosphatases that function as tumor suppressors in various types of human cancer. Here we determined the role of PHLPP in regulating glucose metabolism in colon cancer cells. Knockdown of PHLPP increased the rate of glucose consumption and lactate production, whereas overexpression of PHLPP had the opposite effect. Bioenergetic analysis using Seahorse Extracelluar Flux Analyzer revealed that silencing PHLPP expression induced a glycolytic shift in colon cancer cells. Mechanistically, we found that PHLPP formed a complex with Akt and hexokinase 2 (HK2) in the mitochondrial fraction of colon cancer cells and knockdown of PHLPP enhanced Akt-mediated phosphorylation and mitochondrial localization of HK2. Depletion of HK2 expression or treating cells with Akt and HK2 inhibitors reversed PHLPP loss-induced increase in glycolysis. Furthermore, PHLPP knockdown cells became addicted to glucose as a major energy source in that glucose starvation significantly decreased cancer cell survival. As HK2 is the key enzyme that determines the direction and magnitude of glucose flux, our study identified PHLPP as a novel regulator of glucose metabolism by controlling HK2 activity in colon cancer cells.

## Introduction

Deregulation of cell proliferation in cancer often requires corresponding modifications in the cellular metabolism in order to fuel the increasing need for the biosynthesis of macromolecules including nucleotides, proteins and lipids.^1–3^ Glucose is one of the major carbon sources for many types of cancers. To adapt to the increased demand for the production of energy and metabolic intermediates, cancer cells often upregulate aerobic glycolysis,^4–6^ a phenomenon that was first described by Otto Warburg in 1920s.^7^ Clinically, the increased glycolytic phenotype associated with cancer cells enables the detection of tumor lesions by FDG-PET imaging.^8^ In addition, accumulating evidence has suggested that mitochondrial metabolism is also essential for tumor growth and progression. It has been shown that the mitochondrial function is tightly coupled with increased glucose metabolism in order to produce metabolites not only as building blocks for cell proliferation but also as signaling molecules to control cell signaling (Danvers, MA, USA).^9,10^ For example, Tan *et al*.^11^ reported recently that cancer cells with defected mitochondria show delayed tumor growth in primary lesion and impaired metastatic potential.

Hexokinase (HK) catalyzes the first step of glucose metabolism that is phosphorylating glucose to glucose-6-phosphatase.^12^ There are four isoforms, HK1, 2, 3 and 4, in the mammalian HK family. In addition to their ability to control glycolysis, HK1 and HK2 can associate with mitochondria where they interact with pore-like outer mitochondrial membrane protein voltage-dependent anion channel to regulate apoptosis process.^13,14^ HK1 is ubiquitously expressed in most human tissues, whereas HK2 is the predominant isoform expressed in insulin-sensitive tissues.^15^ Recent studies have shown that overexpression of HK2 promotes cell proliferation and inhibits apoptosis in multiple cancer types including brain, breast, lung, prostate and ovarian cancer.^16–20^ Furthermore, knockout of HK2 inhibits tumor initiation and progression in KRAS-driven lung cancer and ErbB2-driven breast cancer mouse models.^21^ Interestingly, it has been demonstrated that HK2 is phosphorylated by Akt.^22^ This phosphorylation promotes the mitochondrial localization of HK2 to protect cardiomyocytes from metabolic stress-induced apoptosis.^15,22^ However, the functional importance of HK2-mediated glycolysis in colon cancer remains elusive.

PHLPP (PH domain leucine-rich repeat protein phosphatase) belongs to a novel family of Ser/Thr protein phosphatases. There are two isoforms, PHLPP1 and PHLPP2, identified in this family. Serving as negative regulators of several oncogenic protein kinases, including Akt, S6K and RAF1, both PHLPP isoforms function as tumor suppressors by maintaining the balance of cell signaling and homeostasis.^23–28^ Previous studies have shown that loss of PHLPP expression occurs frequently in colon cancer patients^26,29^ and PHLPP loss promotes cell growth and survival *in vitro* and tumorigenesis *in vivo*.^24–26,29,30^ In this study, we investigated the role of PHLPP in modulating glucose metabolism in colon cancer. Our results show that PHLPP negatively regulates glycolysis by inhibiting Akt-dependent phosphorylation and mitochondrial translocation of HK2. Consequently, loss of PHLPP alters mitochondrial respiration and renders cells rely on glucose metabolism for survival.

## Results

### Knockdown of PHLPP increases glucose metabolism in colon cancer cells

**Figure 1:**
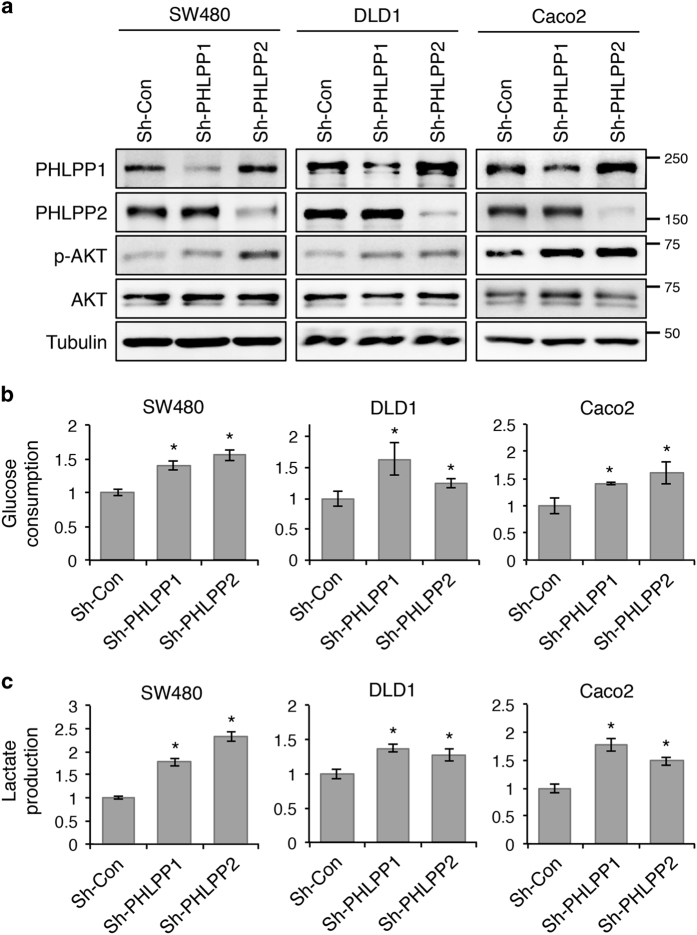
Knockdown of PHLPP isoforms alters glucose utilization. (**a**) Stable control (sh-Con) and PHLPP knockdown (sh-PHLPP1 and sh-PHLPP2) colon cancer cells, including SW480, DLD1 and Caco2, were analyzed for the phosphorylation and expression of Akt, PHLPP1 and PHLPP2 using Western blotting. (**b**) The levels of glucose consumption and (**c**) lactate production were measured in culture medium collected from control and PHLPP knockdown cells. Data represent the mean±S.E.M. (*n*=3, *P<0.05 as determined by two-sample *t*-tests compared with the control cells).

Given the functional connection between Akt and glucose metabolism, and our previous findings that PHLPP directly dephosphorylates and inactivates Akt, we determined if loss of PHLPP expression affects glucose utilization in colon cancer cells. Stable control and PHLPP knockdown cells were cultured in regular growth medium and the levels of glucose consumption and lactate production were measured. Consistent with previous reports, knockdown of either PHLPP isoform resulted in an increase in Akt phosphorylation (Figure 1a). In addition, we found that both glucose consumption and lactate production were significantly increased in multiple PHLPP knockdown colon cancer cell lines suggesting enhanced glycolysis (Figures 1b and c).

**Figure 2:**
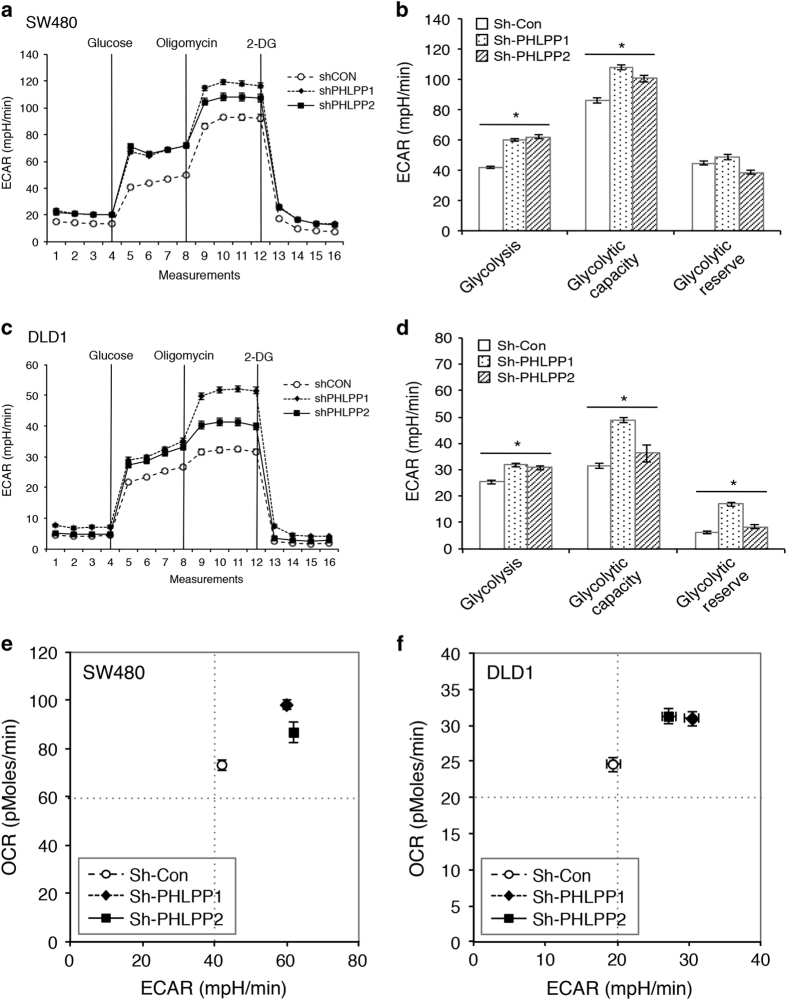
PHLPP regulates glycolysis and mitochondrial respiration in colon cancer cells. (**a**) Representative ECAR measurements obtained from the glycolysis stress test performed in control (sh-Con) and PHLPP knockdown (sh-PHLPP1 and sh-PHLPP2) SW480 cells using the Seahorse XF96 Extracellular Flux analyzer (Agilent). Glucose, oligomycin and 2-deoxyglucose (2-DG) were added at the indicated points. (**b**) Experiments as shown in (**a**) were quantified and ECAR associated with glycolysis, glycolytic capacity and glycolytic reserve was calculated based on the measurements obtained upon the addition of individual compounds. Data represent the mean±S.E.M. (*n*=15, *P<0.05 as determined by two-sample *t*-tests compared with the control cells). (**c**) Representative ECAR measurements obtained from the glycolysis stress test performed in control (sh-Con) and PHLPP knockdown DLD1 cells. (**d**) Experiments and quantification of the data were performed as described above in (**b**). Data represent the mean±S.E.M. (*n*=15, *P<0.05 as determined by two-sample *t*-tests compared with the control cells). (**e**–**f**) Energetic maps of control and PHLPP knockdown SW480 (**e**) and DLD1 cells (**f**). Measurements of ECAR and OCR as described above were quantified. The OCR and ECAR data shown in the map represent the OCR of basal respiration from the Mito stress test and the glycolysis-related ECAR from the glycolysis stress test, respectively (data represent the mean±S.E.M., *n*=15).

To further evaluate the glycolytic phenotype, control and PHLPP knockdown cells were subjected to Seahorse Extracellular Flux analysis to assess the cellular bioenergetic activity. Results from Seahorse glucose stress tests indicated that PHLPP loss significantly increased the extracellular acidification rate (ECAR) associated with both glycolysis and glycolytic capacity in SW480 and DLD1 cells (Figures 2a–d). The glycolytic reserve was also increased in PHLPP knockdown DLD1 cells (Figure 2d). In addition, we found that PHLPP loss altered mitochondrial respiration as determined using Seahorse Mito stress tests. The oxygen consumption rates (OCR) associated with both basal and maximal mitochondrial respiration were significantly unregulated in PHLPP knockdown SW480 and DLD1 cells (Supplementary Figure S1). Furthermore, the bioenergetic measurement of relative utilization of mitochondrial respiration *versus* glycolysis demonstrated that knockdown of either PHLPP isoform enhanced the glycolytic potential as well as the mitochondrial respiration in both cell lines (Figures 2e and f), suggesting that increased glucose utilization is accompanied by increased mitochondrial metabolism. Taken together, these results demonstrate that downregulation of PHLPP promotes the uptake of glucose followed by increased glycolysis and mitochondrial respiration in colon cancer cells.

### PHLPP controls Akt-mediated phosphorylation of HK2

**Figure 3:**
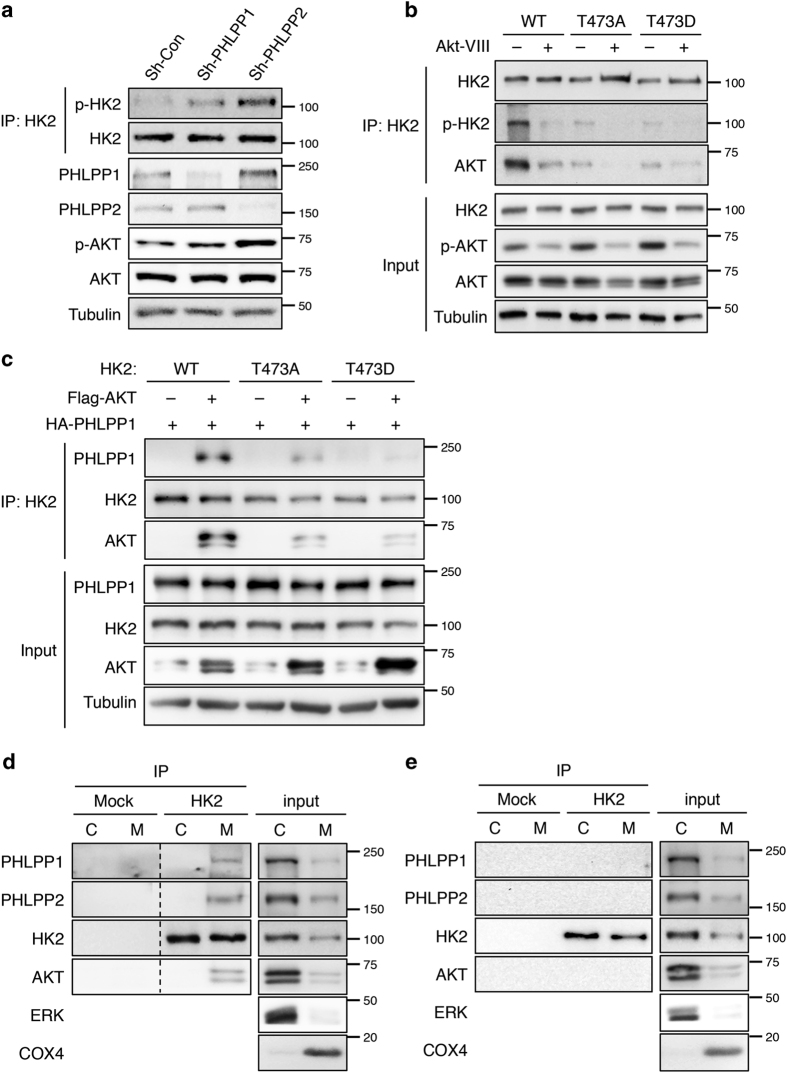
PHLPP regulates HK2 phosphorylation by forming a complex with Akt and HK2. (**a**) Knockdown of PHLPP increases Akt-dependent phosphorylation of HK2. Cell lysates prepared from control and PHLPP knockdown SW480 cells were immunoprecipitated using the HK2 antibody. The phosphorylation of immunoprecipitated HK2 was detected using the phospho-Akt substrate antibody. Cell lysates were probed for the phosphorylation of Akt and the expression of Akt and PHLPP isoforms using Western blotting analysis. (**b**) 293 T cells transfected with WT-, T473A- or T473D-mutant HK2 were lysed and immunoprecipitated with the HK2 antibody. The phosphorylation of HK2 and the presence of endogenous Akt in the immunoprecipitates were detected using the phospho-Akt substrate and Akt antibodies, respectively. The phosphorylation and expression of Akt, and the expression of HK2 and tubulin in the cell lysates were analyzed using Western blotting. (**c**) 293 T cells transfected with HA-PHLPP1 together with WT-, T473A- or T473D-mutant HK2 and Flag-Akt were lysed and immunoprecipitated with the HK2 antibody. The presence of PHLPP1, HK2 and Akt in the immunoprecipitates was detected using corresponding antibodies. The expression of PHLPP, HK2, Akt and tubulin in the cell lysates were analyzed using Western blotting. (**d–e**) SW480 cells cultured under regular growth condition (**d**) or treated with Akt-VIII for 16 h (**e**) were fractionated into the cytosolic (C) and mitochondrial (M) fractions and both fractions were immunoprecipitated with either protein A/G agarose beads alone (mock) or the HK2 antibody. The amount of proteins from each fraction in the input represent 10% of total lysates used for immunoprecipitation. The presence of PHLPP1, PHLPP2, HK2 and Akt in the immunoprecipitates were detected using corresponding antibodies. ERK and COX4 were used as markers of cytosolic and mitochondrial proteins, respectively.

Inhibition of mitochondrial respiration often leads to increased glycolysis at the expense of oxidative phosphorylation. However, since downregualtion of PHLPP resulted in increased levels of glycolysis and mitochondrial respiration rather than switching cells from oxidative phosphorylation to glycolysis, we reasoned that PHLPP likely targets a pivotal regulator of glycolysis directly. HK2, a substrate of Akt, has been shown to promote tumor growth via its ability to control glycolysis.^21^ Here we investigated whether knockdown of PHLPP alters HK2 phosphorylation. To analyze Akt-mediated phosphorylation of HK2, HK2 was immunoprecipitated from control and PHLPP knockdown SW480 cells and Akt-mediated phosphorylation of HK2 was detected using the phospho-Akt-substrate antibody. Indeed, HK2 phosphorylation was markedly increased in both PHLPP1 and PHLPP2 knockdown cells, whereas the expression of total HK2 remained unchanged (Figure 3a). Consistently, the phosphorylation of Akt was also elevated in PHLPP knockdown cells indicating increased activation (Figure 3a). Moreover, we found that Akt-mediated phosphorylation of HK2 was largely increased in PHLPP knockout mouse embryonic fibroblast (MEF) cells, which coincided with elevated levels of glucose consumption and lactate production (Supplementary Figure S2). Similar as in colon cancer cells, Seahorse bioenergetic measurements showed that PHLPP knockout MEF cells have increased levels of glycolysis and mitochondrial respiration (Supplementary Figure S2). Together, these results suggest that PHLPP may regulate glycolysis by controlling the phosphorylation of HK2.

Previous studies have shown that HK2 is phosphorylated by Akt at T473 site.^22^ To further define the specificity of Akt-mediated phosphorylation of HK2, we expressed wild-type (WT), and phosphorylation mutants of HK2 (including the phosphorylation-deficient T473A and phospho-mimetic T473D) in 293T cells and analyzed the levels of phosphorylation using the phospho-Akt substrate antibody. As shown in Figure 3b, the phosphorylation was detected only in WT HK2 under the control condition, whereas the phospho-Akt substrate antibody failed to recognize both HK2 mutants and WT HK2 when treated with Akt inhibitor (Akt-VIII), thus confirming that HK2 is phosphorylated by Akt at the T473 site in cells. Intriguingly, HK2 was found to interact with Akt in a phosphorylation-dependent manner as both HK2 mutants and dephosphorylated HK2 in Akt-VIII-treated cells did not co-immunoprecipitated with Akt (Figure 3b).

Furthermore, as PHLPP in known to interact with Akt as well,^24,25^ we investigated if PHLPP, Akt and HK2 form a functional complex. Results from co-immunoprecipitation experiments showed that WT HK2 did not bind PHLPP by itself, whereas co-expression of Akt markedly increased the interaction between WT HK2 and PHLPP in 293T cells (Figure 3c). Both HK2 mutants interacted with Akt and PHLPP with much reduced affinity (Figure 3c). In addition, we analyzed the nature of the endogenous PHLPP–Akt–HK2 complex in colon cancer cells. SW480 cells were fractionated into the mitochondrial and cytosolic fractions and the presence of PHLPP, Akt and HK2 were detected in both fractions (Figure 3d). Despite that only a small percentage of total cellular PHLPP, Akt and HK2 proteins were found localized to the mitochondrial fraction, a PHLPP–Akt–HK2 protein complex was readily detected in the mitochondria but not in the cytosol (Figure 3d). Consistent with our finding that Akt inhibitor disrupts the interaction between HK2 and Akt, the mitochondrial PHLPP–Akt–HK2 complex was dissociated in cells treated with Akt inhibitor (Figure 3e). However, as Akt activity is required for maintaining PHLPP protein stability,^31,32^ we were unable to determine if silencing Akt disrupts PHLPP–HK2 interaction directly as Akt loss significantly reduced the total expression of PHLPP. Collectively, our results are consistent with the notion that Akt functions as a bridge to facilitate the formation of PHLPP–Akt–HK2 complex and Akt-mediated phosphorylation of HK2 at T473 residue is required for its association with the complex.

### PHLPP regulates mitochondrial localization of HK2

**Figure 4:**
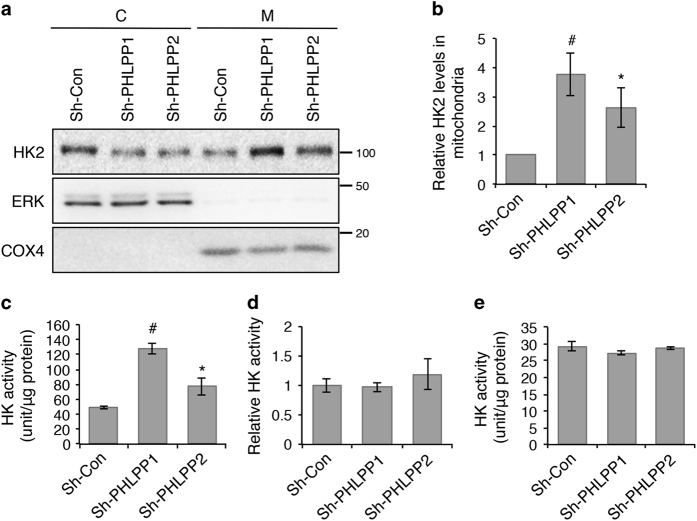
Knockdown of PHLPP promotes mitochondrial association of HK2. (**a**) Control and PHLPP knockdown SW480 cells were fractionated into cytosolic (C) and mitochondrial (M) fractions. The distribution of HK2 in each fraction was analyzed using Western blotting. ERK and COX4 were used as markers of cytosolic and mitochondrial proteins, respectively. (**b**) The relative expression of HK2 in the mitochondrial fraction was quantified by normalizing to COX4 and compared with the control cells. Data represent mean±S.E.M. (*n*=5, #*P*<0.01 and *P<0.05 as determined by two-sample *t*-tests compared with the control cells). (**c**) The HK activity was determined in the mitochondrial fraction of the control and PHLPP knockdown SW480 cells. The activity was normalized to the amount of total proteins in the assay. Data represent mean±S.E.M. (*n*=3, #*P*<0.01 and *P<0.05 as determined by two-sample *t*-tests compared with the control cells). (**d**) The HK activity was determined in the mitochondrial fraction as described in (**c**). The mitochondrial protein lysates used for enzyme activity assays were analyzed by Western blotting to determine the level of HK2 in each assay. The relative HK activity was obtained by normalizing to the amount of HK2 detected by the HK2 antibody. Data represent mean±S.E.M. No statistical difference was found among cell lines. (**e**) The HK activity was determined in the whole-cell lysates of the control and PHLPP knockdown SW480 cells. The activity was normalized to the amount of total proteins in the assay. Data represent mean±S.E.M. No statistical difference was found among cell lines.

To determine if PHLPP expression regulates the cellular localization of HK2, control and PHLPP knockdown SW480 cells were fractionated into the mitochondrial and cytosolic fractions. We found that downregulation of PHLPP resulted in a significant increase in the amount of HK2 associated with the mitochondrial fraction (Figures 4a and b). This is consistent with previous reports that Akt-mediated phosphorylation of HK2 promotes its translocation to the mitochondria.^13,22^ Furthermore, the HK activity associated with the mitochondrial fraction was significantly increased in PHLPP knockdown cells when normalized to the amount of total mitochondrial proteins (Figure 4c). However, when normalizing to the amount of HK2 detected in the mitochondria, the relative HK activity was not altered in PHLPP knockdown cells (Figure 4d). As a control, the total HK activity in PHLPP knockdown cells also remained unchanged (Figure 4e). Taken together, our results suggest that PHLPP loss increases the level of HK2 activity associated with the mitochondria by promoting mitochondrial association of HK2 rather than directly altering HK2 enzyme activity.

### Overexpression of PHLPP inhibits glycolysis and HK phosphorylation

**Figure 5:**
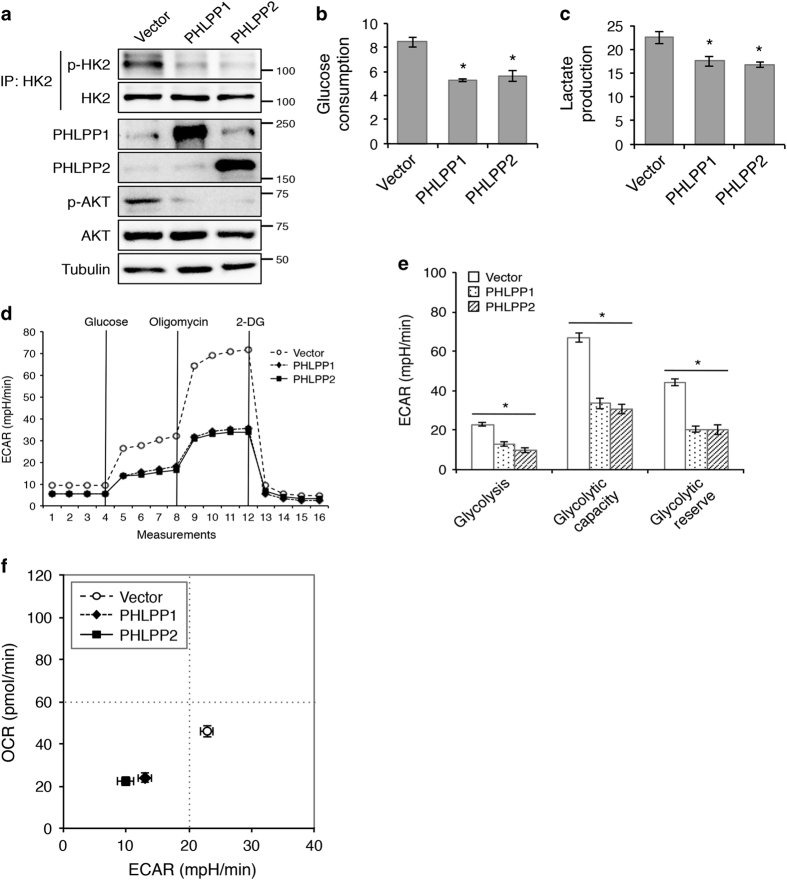
Overexpression of PHLPP inhibits glycolysis and mitochondrial respiration via dephosphorylation of HK2. (**a**) Stable control and PHLPP overexpressing SW480 cells were analyzed for Akt-mediated HK2 phosphorylation. Cell lysates were immunoprecipitated using the HK2 antibody. The phosphorylation of immunoprecipitated HK2 was detected using the phospho-Akt substrate antibody. The cell lysates were probed for the phosphorylation of Akt and the expression of Akt and PHLPP isoforms using Western blot analysis. (**b**) The levels of glucose consumption and (**c**) lactate production were measured in culture medium collected from control and PHLPP overexpressing cells. Data represent the mean±S.E.M. (*n*=3, *P<0.05 as determined by two-sample *t*-tests compared with the control cells). (**d**) Representative measurements obtained from the glycolysis stress test performed in control (vector) and PHLPP overexpressing cells using the Seahorse XF96 Extracellular Flux analyzer (Agilent). (**e**) Experiments as shown in (**d**) were quantified and ECAR associated with glycolysis, glycolytic capacity and glycolytic reserve was calculated based on the measurements obtained upon the addition of individual compounds. Data represent the mean±S.E.M. (*n*=15, *P<0.05 as determined by two-sample *t*-tests compared with the control cells). (**f**) A representative energetic map of control and PHLPP overexpressing cells. The OCR and ECAR data shown in the map represent the OCR of basal respiration from the Mito stress test and the glycolysis-related ECAR from the glycolysis stress test, respectively (data represent the mean±S.E.M., *n*=15).

To investigate the effect of PHLPP overexpression on cellular metabolism, we generated stable SW480 cells overexpressing PHLPP isoforms and examined the phosphorylation of HK2. In contrast to PHLPP knockdown cells, the phosphorylation of HK2 was largely decreased in PHLPP overexpressing cells as detected by the phospho-Akt substrate antibody (Figure 5a). Consistent with previous reports,^26,29^ PHLPP overexpression also resulted in a decrease in Akt phosphorylation (Figure 5a). Next, we measured glucose consumption and lactate production in PHLPP overexpressing SW480 cells. Overexpression of either PHLPP isoform significantly decreased both glucose consumption and lactate production indicating reduced glycolysis (Figures 5b and c). Furthermore, Seahorse glycolysis stress tests revealed that PHLPP overexpression significantly decreased ECAR associated with glycolysis, glycolytic capacity as well as glycolytic reserve (Figures 5d and e). In addition, OCR associated with basal and maximal mitochondrial respiration and ATP turnover were significantly inhibited in PHLPP overexpressing cells as indicated by Seahorse Mito stress test (Supplementary Figure S3). Finally, bioenergetic measurements showed that overexpression of either PHLPP isoform inhibited both glycolysis and mitochondrial respiration in colon cancer cells (Figure 5f). Taken together with results obtained in PHLPP knockdown cells, we show that PHLPP has an important role in regulating the balance of overall bioenergetics in colon cancer.

### HK2 regulates glucose metabolism in colon cancer cells

**Figure 6:**
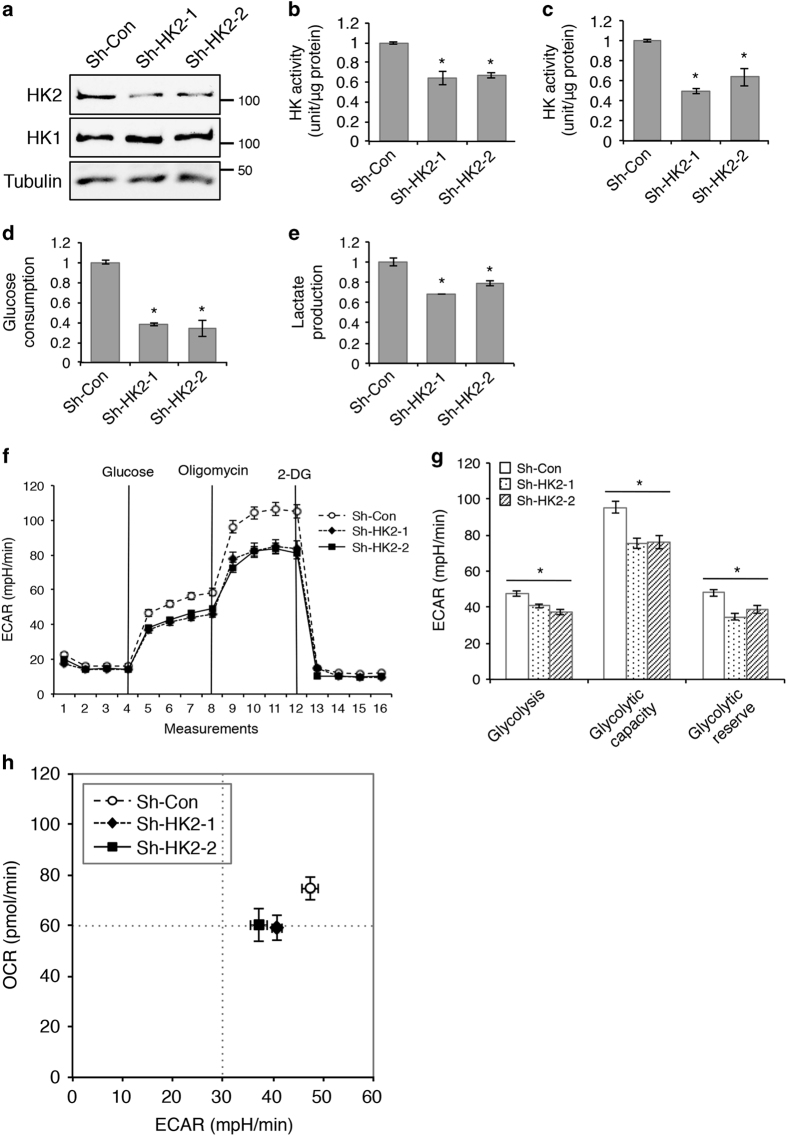
Knockdown of HK2 inhibits glycolysis and mitochondrial respiration in colon cancer cells. (**a**) Stable control and two HK2 knockdown (sh-HK2-1 and sh-HK2-2) SW480 cells were analyzed for HK2 and HK1 expression using Western blotting. (**b**) The HK activity was determined in the whole-cell lysates and (**c**) the mitochondrial fraction of control and HK2 knockdown cells. Data represent mean±S.E.M. (*n*=3, *P<0.05 as determined by two-sample *t*-tests compared with the control cells). (**d**) The levels of glucose consumption and (**e**) lactate production were measured in culture medium collected from control and HK2 knockdown cells. Data represent the mean±S.E.M. (*n*=3, *P<0.05 as determined by two-sample *t*-tests compared with the control cells). (**f**) Representative measurements obtained from the glycolysis stress test performed in control and HK2 knockdown cells using the Seahorse XF96 Extracellular Flux analyzer (Agilent). (**g**) Experiments as shown in (**f**) were quantified and ECAR associated with glycolysis, glycolytic capacity and glycolytic reserve was calculated based on the measurements obtained upon the addition of individual compounds. Data represent the mean±S.E.M. (*n*=15, *P<0.05 as determined by two-sample *t*-tests compared with the control cells). (**h**) A representative energetic map of control and HK2 knockdown cells. The OCR and ECAR data shown in the map represent the OCR of basal respiration from the Mito stress test and the glycolysis-related ECAR from the glycolysis stress test, respectively (data represent the mean±S.E.M., *n*=15).

To determine the functional effect of HK2 on regulating glucose metabolism directly, we created stable HK2 knockdown SW480 cells using two different shRNA targeting sequences, and knockdown of HK2 had no effect on HK1 expression (Figure 6a). The HK activity was effectively decreased by ~40–50% in total cell lysates and in mitochondrial fractions in the knockdown cells (Figures 6b and c). Consequently, the levels of glucose consumption and lactate production were significantly reduced (Figures 6d and e). Results from Seahorse metabolic measurements showed that ECAR associated with glycolysis, glycolytic capacity and glycolytic reserve were all significantly decreased in HK2 knockdown cells (Figures 6f and g). Similarly, OCR associated with basal and maximal mitochondrial respiration were decreased as well (Supplementary Figure S4). The bioenergetic measurements revealed that knockdown of HK2 reduced the glycolytic potential as well as mitochondrial respiration in colon cancer cells (Figure 6h). These findings are reminiscent of results obtained in PHLPP overexpressing cells (Figure 5), thus supporting the notion that PHLPP regulates glucose metabolism by controlling HK2 activity.

### Inhibition of Akt or HK2 activity reverses PHLPP loss-induced increase in glycolysis

**Figure 7:**
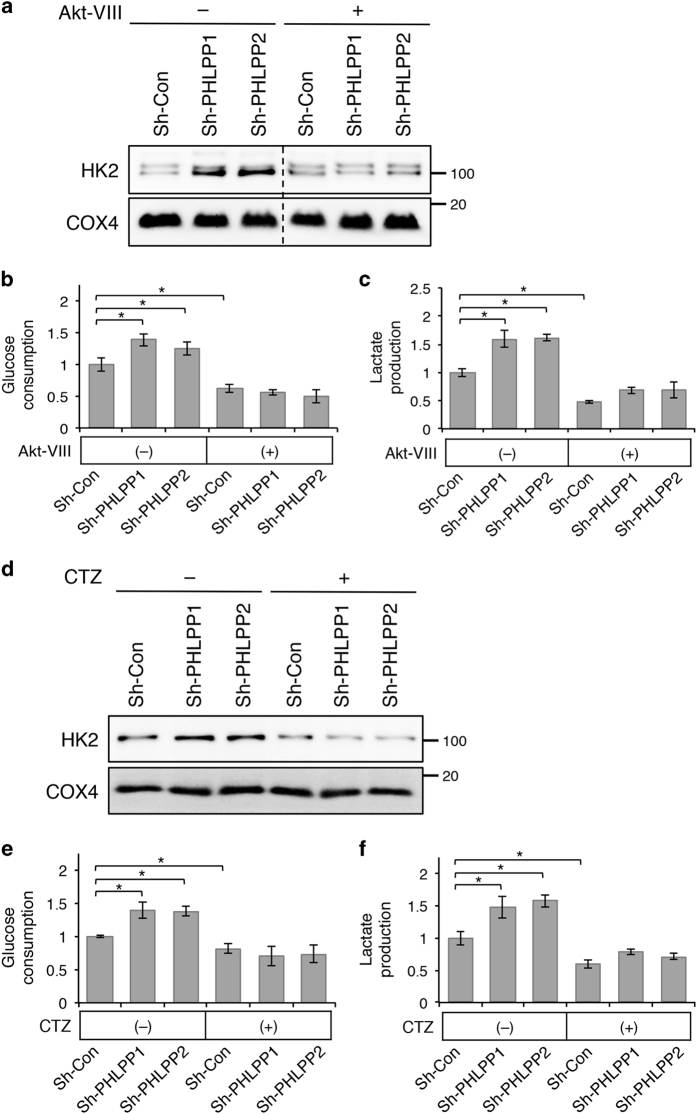
Inhibition of Akt activity or HK2 mitochondrial localization antagonizes PHLPP loss-induced increases in glycolysis. (**a**) Stable control and PHLPP knockdown SW480 cells were treated with DMSO or Akt inhibitor VIII for 16 h in regular growth medium. Mitochondrial fractions were prepared and analyzed for the levels of HK2 using Western blotting. COX4 was used as a marker for mitochondrial proteins. (**b**) The levels of glucose consumption and (**c**) lactate production were measured in culture medium collected from the cells as treated in (**a**). Data represent the mean±S.E.M. (*n*=3, *P<0.05 as determined by two-sample *t*-tests compared with control cells under basal condition). (**d**) Stable control and PHLPP knockdown SW480 cells were treated with DMSO or CTZ for 16 h in regular growth medium. Mitochondrial fractions were prepared and analyzed for the levels of HK2 using Western blotting. COX4 was used as a marker for mitochondrial proteins. (**e**) The levels of glucose consumption and (**f**) lactate production were measured in culture medium collected from the cells as treated in (**d**). Data represent the mean±S.E.M. (*n*=3, *P<0.05 as determined by two-sample *t*-tests compared with control cells under basal condition).

As we found that knockdown of PHLPP increases mitochondrial localization of HK2 by promoting Akt-dependent phosphorylation, we next determined the effect of Akt inhibition on PHLPP-mediated regulation of glycolysis. Stable control and PHLPP knockdown SW480 cells were treated with Akt inhibitor and the expression of HK2 in mitochondrial fractions was examined. Similar as shown in Figure 4a, the amount of HK2 found in the mitochondrial fraction was increased in PHLPP knockdown cells; however, this increase in mitochondrial HK2 was effectively blocked by treating cells with Akt-VIII (Figure 7a). Functionally, this reduced mitochondrial localization of HK2 coincided with changes in glucose consumption and lactate production, in which Akt-VIII treatment inhibited glucose consumption and lactate production in both control and PHLPP knockdown cells and eliminated the increase in glycolysis induced by PHLPP loss (Figures 7b and c). Moreover, we examined if dissociation of HK2 from the mitochondria affects PHLPP-mediated regulation of glycolysis. Cells were treated with clotrimazole (CTZ), a previously characterized HK2 inhibitor that functions by dissociating HK2 from mitochondria.^13^ CTZ treatment led to a significant detachment of HK2 from the mitochondria in both control and PHLPP knockdown SW480 cells (Figure 7d). As a consequence, the increase in glucose consumption and lactate production observed in PHLPP knockdown cells was eliminated (Figures 7e and f). Collectively, our results indicate that inhibition of Akt activity or dissociation of HK2 from the mitochondria precludes cells from responding to PHLPP downregulation.

### Knockdown of HK2 attenuates the effect of PHLPP loss on glycolysis

**Figure 8:**
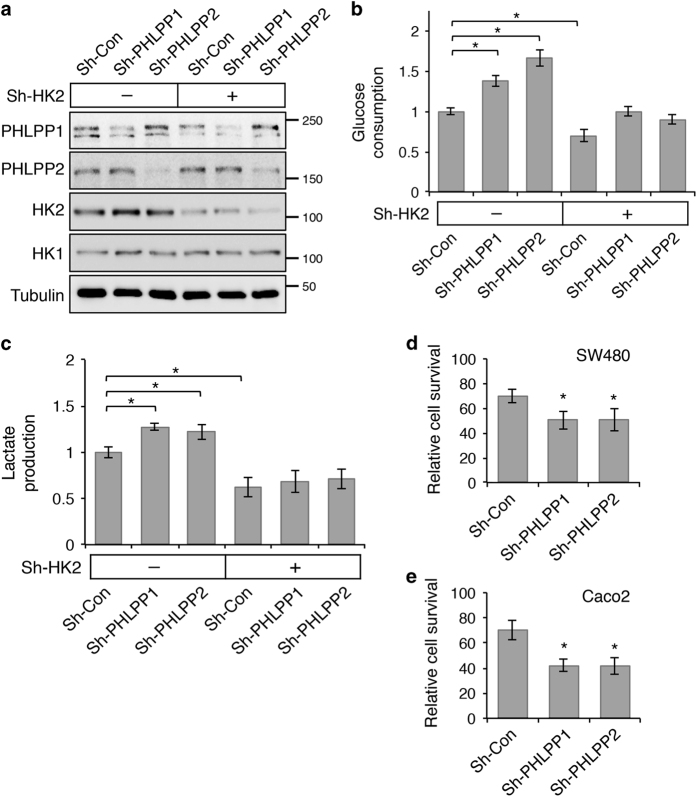
Knockdown of HK2 blocks PHLPP loss induced increases in glycolysis. (**a**) Stable control and PHLPP knockdown SW480 cells were infected with shRNA lentiviruses targeting HK2 (sh-HK2-1). The expression of HK1, HK2 and PHLPP isoforms was detected using Western blot analysis. (**b**) The levels of glucose consumption and (**c**) lactate production were measured in culture medium collected from the cells as described in (**a**). Data represent the mean±S.E.M. (*n*=3, *P<0.05 as determined by two-sample *t*-tests compared with control cells). The control and PHLPP knockdown SW480 (**d**) and Caco2 (**e**) cells were cultured in either the high-glucose or the low-glucose medium for 48 h. The relative cell survival was determined by comparing the cell growth under the low-glucose condition with that of the high-glucose condition. Data represent the mean±S.E.M. (*n*=6, *P<0.05 as determined by two-sample *t*-tests compared with the control cells).

To further determine the functional contribution of HK2 downstream of PHLPP, HK2 expression was depleted in PHLPP knockdown cells (Figure 8a). Consistent with results shown earlier, knockdown of either PHLPP isoform significantly increased, whereas silencing HK2 expression decreased glucose consumption and lactate production in SW480 cells (Figures 8b and c). Importantly, PHLPP loss-induced increase in glycolysis was abolished in cells where HK2 was also knocked down (Figures 8b and c), thus suggesting that HK2 functions downstream of PHLPP to regulate glycolysis.

Furthermore, we determined the functional implication of PHLPP-mediated regulation of glucose metabolism in colon cancer cells. Control and PHLPP knockdown cells were cultured in high- or low-glucose medium for 48 h, and the relative cell survival was determined by comparing the extent of cell growth under the low-glucose condition to that of the high-glucose condition. Interestingly, silencing PHLPP expression rendered both SW480 and Caco2 cells more sensitive to glucose restriction in that the relative cell survival was significantly decreased in PHLPP knockdown cells when cultured under the low-glucose condition (Figures 8d and e). Taken together, our data indicate that downregulation of PHLPP allows the cancer cells to preferentially utilize glucose as a metabolic substrate to support cell growth and survival.

## Discussion

Cancer cells are rapidly dividing cells that have increased demands for energy and macromolecules. To cope with these elevated requirements cancer cells undergo major metabolic modifications.^2,33^ Emerging evidence suggests that activated oncogenes and inactivated tumor suppressors may drive tumorigenesis by rewiring cellular metabolic pathways. Overall, metabolic reprogramming allows cancer cells to utilize limited amount of nutrients available in the tumor microenvironment to overcome metabolic stresses.^2^ Our study here has identified a novel role of PHLPP in regulating cellular metabolism in colon cancer cells. We show that loss of PHLPP expression promotes glycolysis and mitochondrial respiration by upregulating Akt-dependent phosphorylation and mitochondrial translocation of HK2. Importantly, our findings that PHLPP loss induced upregulation of glycolysis can be abolished by inhibition of Akt, dissociation of HK2 from the mitochondria or knockdown of HK2 indicate that PHLPP regulates glucose metabolism through a PHLPP/Akt/HK2 axis in colon cancer cells.

Previous studies have identified PHLPP as a tumor suppressor in several types of cancer. Loss of PHLPP expression has been linked to increased cell proliferation, decreased apoptosis and increased ability of cancer cells to migrate and invade by inducing epithelial–mesenchymal transition.^24–26,28,29,34–37^ Interestingly, it has been shown that PHLPP inhibits protein synthesis by directly dephosphorylating and inactivating S6K, a key regulator of protein translation, and PHLPP loss increases the rate of cap-dependent protein synthesis.^27^ This increased biomass synthesis is likely coupled with heightened demands for ATP and metabolic intermediates. We show in this study that PHLPP depletion allows cancer cells to upregulate glucose metabolism and mitochondrial respiration. The mitochondrial respiration is likely activated because the mitochondria are responsible for further processing the intermediate metabolites produced by the glycolytic pathway. Overall, the metabolic alterations induced by PHLPP loss may provide the necessary building blocks needed for growing cell mass. In addition, consistent with our previous report that PHLPP expression is significantly downregulated under hypoxia,^38^ our findings here suggest that PHLPP loss may contribute to altered glucose metabolism in response to hypoxic stress in colon cancer.

Results from our study identify HK2 as a major effector of PHLPP-mediated regulation of glycolysis. HK2 has been shown to be the only HK isoform that is regulated by Akt-dependent protein phosphorylation.^15,22^ Phosphorylation of HK2 at T473 site promotes mitochondrial binding of HK2, which facilitates the coupling of glycolysis and oxidative phosphorylation through preferential access of HK2 to ATP generated by mitochondria.^39^ Indeed, although the amount of HK2 localized in the mitochondrial fraction is <5% of total HK2 expressed based on Western blotting analysis, the HK activity associated with the mitochondria is at least five times higher than that in the cytosol in SW480 cells (data not shown). Thus, the mitochondria-localized HK2 may have a functionally more important role. Using the phospho-Akt substrate antibody and phosphorylation site mutations of HK2, we demonstrate that PHLPP negatively regulates the phosphorylation of HK2 at T473 site and subsequently the mitochondrial association of HK2. In our study, we also find that purified phosphatase domains of PHLPP can dephosphorylate HK2 directly *in vitro* (data not shown) raising the possibility that PHLPP may function as a phosphatase of HK2 in addition to its ability to dephosphorylate Akt. However, as Akt is required for bringing PHLPP to the PHLPP–Akt–HK2 complex at the mitochondria, we interpret our data as suggesting that PHLPP-mediated regulation of HK2 phosphorylation likely occurs within the PHLPP–Akt–HK2 complex where PHLPP has a direct access to its substrate Akt, and potentially HK2. Regardless of a direct or indirect mechanism of action, our study identifies a signaling complex that is consisted of both a positive and a negative regulator of HK2 at the mitochondria; and the formation of this localized complex may allow for rapid adjustments of HK2 activity based on shifting metabolic requirements.

It is intriguing that the HK2-T473D mutant fails to bind Akt efficiently. It has been shown previously that the amount of mitochondrial localization of HK2-T473D is similar as that of the phosphorylated WT HK2.^22^ However, HK2 is known to localize to the mitochondria by binding to voltage-dependent anion channel on the outer membrane of mitochondria,^13,15,40^ where it may also interact with Akt. It is possible that the T473D mutation allows HK2 to bind voltage-dependent anion channel with higher affinity as it becomes more resistant to glucose-6-phosphate-induced dissociation;^22^ however, the Glu substitution is not sufficient to mimic a phosphate group in mediating the interaction with Akt. Future studies are needed to determine if the PHLPP–Akt–HK2 complex is anchored to the mitochondria via binding to voltage-dependent anion channel.

In summary, results from our study provide strong evidence suggesting that PHLPP has an important role in regulating glucose metabolism by controlling Akt and HK2 function in colon cancer cells. As PHLPP is lost in a majority of colon cancer patients^26,29^ and PHLPP loss renders cancer cells sensitive to glucose restriction-induced cell death, selectively targeting HK2 (such as using agents that dissociate HK2 from mitochondria) may provide a novel treatment option for colon cancer patients.

## Materials and Methods

### Antibodies and reagents

Antibodies against PHLPP1 and PHLPP2 were purchased from Proteintech (Rosemont, IL, USA) and Bethyl Laboratory (Montgomery, TX, USA), respectively. The HK1, HK2, ERK, Akt, phosphor-Akt (Ser473) and phospho-Akt-substrate (RXXS*/T*, 110B7E) antibodies were from Cell Signaling. The HK2 (used for immunoprecipitation) and COX4 antibodies were from Santa Cruz Biotechnology (Dallas, TX, USA). The expression plasmids for HA-tagged PHLPP1 and PHLPP2 have been described in previous studies.^24,25,29,38^ CTZ was obtained from Sigma-Aldrich (St Louis, MO, USA) and used at 20 *μ*M and Akt inhibitor VIII was from Millipore (Billerica, MA, USA) and used at 1 *μ*M for the treatment of cells. The coding sequence of mouse HK2 was amplified using PCR and subcloned into pcDNA4-Myc/His vector. Mutant HK2 expression plasmids including T473A and T473D were generated using QuickChange site-directed mutagenesis kit (Agilent, Santa Clara, CA, USA).

### Cell culture

Human colon cancer cell lines, including SW480, DLD1 and Caco2, and 293 T and MEF cells were cultured in DMEM supplemented with 10% fetal bovine serum (FBS, Sigma-Aldrich) and 1% penicillin–streptomycin. Stable PHLPP and HK2 knockdown cells were generated by lentivirus-based shRNAs as previously described.^26,29,38^ The shRNA targeting sequences for HK2 are as the following: 5′- CCGTAACATTCTCATCGATTT-3′ (#1), and 5′- ACTGAGTTTGACCAGGAGATT-3′ (#2). Overexpression of PHLPP isoforms in colon cancer cells was achieved by infecting cells with retrovirus encoding HA-tagged PHLPP1 or PHLPP2 and selecting with puromycin. MEF cells were isolated from WT, Phlpp1^–/–^ or Phlpp2^–/–^ mouse embryos at day 14 of gestation and immortalized by knocking down p53 expression.^26,41^ Transient transfection of 293T cells with the expression plasmids encoding HA-tagged PHLPP1 and PHLPP2 was carried out using PEI-mediated method.

### Immunoprecipitation and Western blot analysis

Cells were washed in ice-cold PBS three times and lysed with lysis buffer (50 mM Na_2_HPO_4_, pH 7.4, 1 mM sodium pyrophosphate, 20 mM NaF, 2 mM EDTA, 2 mM EGTA, 1% Triton X-100, 1 mM DTT, 200 mM benzamidine, 40 mg/ml leupeptin, 200 mM PMSF) and the detergent-solublized cell lysates were obtained after centrifugation for 5 min at 16 000×*g* at 4 °C.^26,29,38^ Equal amounts of solublized cell lysates were resolved by SDS-PAGE and subjected to Western blot analysis. To examine the phosphorylation status of HK2 and the interaction between HK2 and PHLPP, the solublized cell lysates were incubated with the HK2 antibody and protein A/G agarose beads (Pierce) at 4 °C for overnight. The beads were washed three times with lysis buffer and the immunoprecipitated proteins were analyzed by SDS-PAGE and Western blotting.

### Mitochondrial fractionation

Cells washed with ice-cold PBS were lysed in mitochondrial lysis buffer (250 mM sucrose, 20 mM HEPES, pH 7.4, 20 mM NaF, 10 mM KCl, 1.5 mM MgCl_2_, 1 mM EDTA, 1 mM EGTA, 1 mM DTT, 200 mM benzamidine, 40 mg/ml leupeptin and 200 mM PMSF). After brief sonication, samples were centrifuged at 1000×*g* for 10 min at 4 °C to remove cell debris and nuclei. The supernatant was subsequently centrifuged again at 13 000 ×*g* for 20 min at 4 °C and the resulting supernatant was saved as the cytosol fraction. The pellet was washed twice with mitochondrial lysis buffer, dissolved in lysis buffer and saved as the mitochondria fraction. The mitochondria and cytosol fractions were resolved by SDS-PAGE and subjected to Western blot analysis. Separation of COX4 and ERK into the mitochondria and cytosol fraction, respectively, was used as the control for the fractionation experiments.

### Measurements of glucose consumption and lactate production

Cells were seeded in six-well plate at the density of 5×10^5^ cells per well in 1 ml DMEM growth medium. A blank well without cells was included as the control. After 16 h incubation, glucose and lactate concentrations in the culture medium were measured using the Glucose Assay Kit and l-Lactate Assay Kit (Biovison, Milpitas, CA, USA), respectively. The levels of glucose consumption and lactate production were determined by subtracting the concentration of glucose or lactate in the control medium from that in the sample medium. The numbers of cells in each well were counted and used for normalizing the data.

### Hexokinase activity assay

The HK activity was determined using the Hexokinase Assay Kit per manufacturer’s protocol (Biomedical Research Service Center, SUNY, Buffalo, NY, USA). Briefly, 1×10^6^ cells washed in ice-cold PBS were resuspended in 100 *μ*l of HK cell lysis solution. The cell lysates were centrifuged for 3 min at 13 000×*g* at 4 °C and the resulting supernatant was used in the HK activity assay. In experiments where the mitochondrial HK activity was measured, cells were first fractionated into mitochondria and cytosol fractions and only the mitochondria fraction was used in the HK activity assay. The reactions were carried out in 96-well plates at 37 °C for 1 h and the HK activity (expressed as IU/l) was calculated based on the absorbance at OD_492_ and normalized to the amount of proteins in each sample or to the levels of HK2 as detected by Western blotting.

### Seahorse extracellular flux analysis

The Seahorse XF96 Extracellular Flux Analyzer (Agilent) was used to measure cellular respiration activity in colon cancer cells. Cells were seeded at the density of 3×10^4^ cells per well in a XF96 plate ~12 h before the measurement. The glycolysis and mitochondrial stress test were performed per manufacturer’s protocol. The relative levels of non-glycolytic acidification, glycolysis, glycolytic capacity and glycolytic reserve were calculated based on OCR and ECAR data obtained in the glycolysis stress tests, whereas the relative levels of non-mitochondrial, basal, maximal and ATP production-related respiration were calculated based on the Mito stress tests using Seahorse Wave software (Agilent) for XF analyzers.

### Cell viability assay

Equal numbers of control and PHLPP knockdown cells (20 000 cells per well) were seeded onto 24-well plates and cultured overnight in regular growth medium (DMEM containing 4.5 g/l glucose plus 10% FBS). Cells were then switched to low-glucose growth medium (DMEM containing 1 g/l glucose plus 10% FBS) or maintained in regular growth medium for additional 48 h. At the end of the experiments, the cells were fixed and stained with 0.5% crystal violet in 20% methanol for 30 min. After washing with water, the stained cells were dissolved in 1% SDS and absorbance at OD_570_ was measured.^29^ The relative cell survival was calculated by comparing the OD_570_ values obtained under the low-glucose condition with those of the regular growth medium.
