# Supplementary material for: PHLPP regulates hexokinase 2-dependent glucose metabolism in colon cancer cells
Source: Cell Death Discov. 2017 Jan 23;3:16103–. doi: 10.1038/cddiscovery.2016.103 (PMC5253420; doi:10.1038/cddiscovery.2016.103)

**Supplemental Figures**

**Figure S1. Knockdown of PHLPP alters mitochondrial respiration in colon cancer cells.** (**a**) Representative OCR measurements obtained from the Mito tress test performed in control (sh-Con) and PHLPP knockdown (sh-PHLPP1 and sh-PHLPP2) SW480 cells using the Seahorse XF96 Extracellular Flux analyzer. Oligomycin, FCCP and antimycin A/rotenone (Anti-A) were added at the indicated points. (**f**) Experiments as shown in (**e**) were quantified and the relative levels of OCR associated with basal and maximal respiration and ATP turnover were calculated based on the measurements obtained upon the addition of individual compounds. Data represent the mean ± SEM (n=15, * p<0.05 as determined by two-sample t-tests compared to the control cells). (**g**) Representative OCR measurements obtained from the Mito tress test performed in control (sh-Con) and PHLPP knockdown (sh-PHLPP1 and sh-PHLPP2) DLD1 cells. (**h**) Experiments and quantification of the data were performed as described above in (**f**). Data shown on the right represent the mean ± SEM (n=15, * p<0.05 as determined by two-sample t-tests compared to the control cells).

**
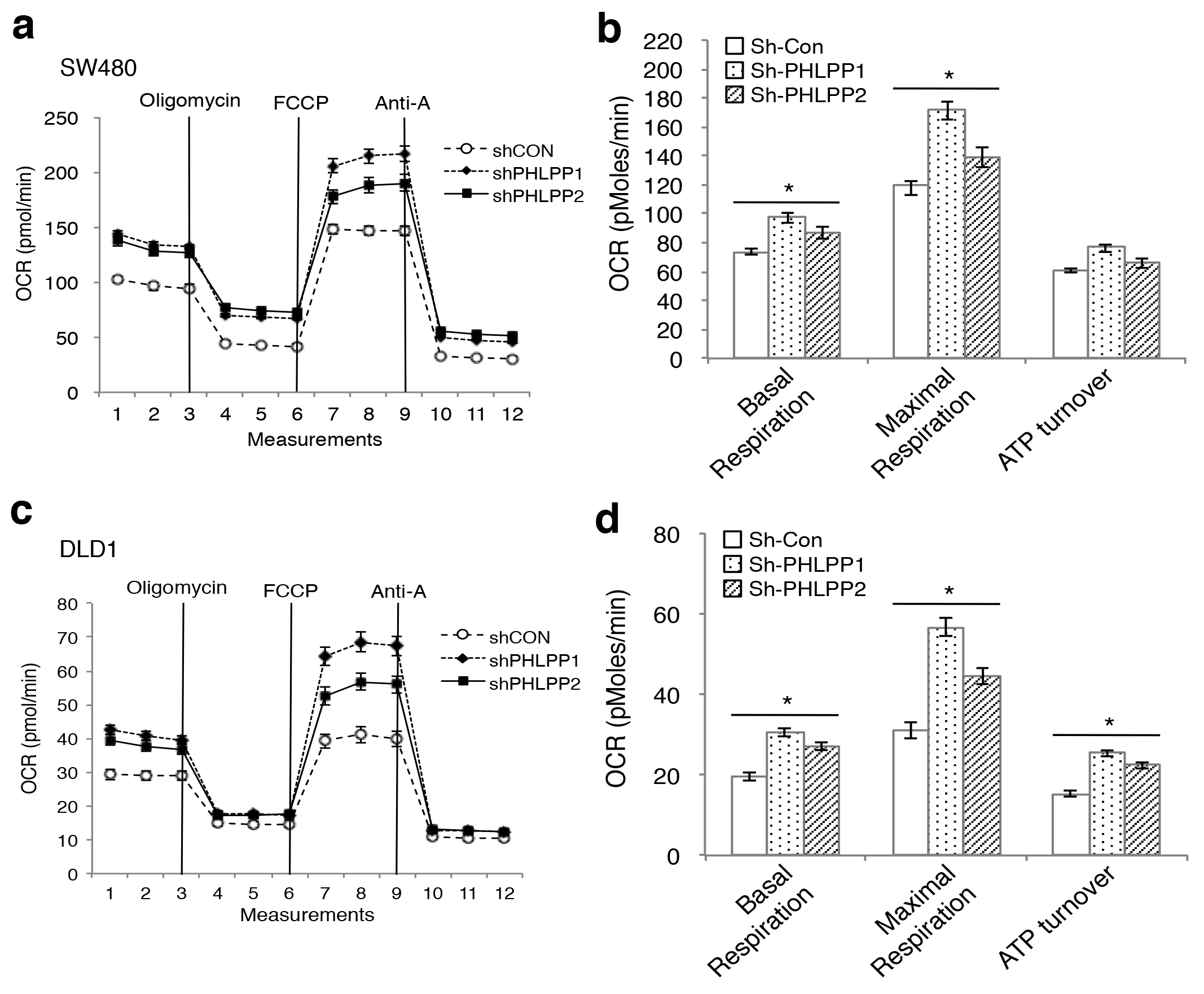
**

**Figure S2. Loss of PHLPP promotes glycolysis in MEF cells.** (**a**) Knockout of PHLPP increases Akt-dependent phosphorylation of HK2 in MEF cells. Cell lysates prepared from wild-type (WT) and PHLPP knockout (Phlpp1^–/–^ and Phlpp2^–/–^) MEF cells were immunoprecipitated using the HK2 antibody. The phosphorylation of immunoprecipitated HK2 was detected using the phospho-Akt substrate antibody. The cell lysates were probed for the phosphorylation of Akt and the expression of Akt and PHLPP isoforms using Western blot analysis. (**b**) The levels of glucose consumption and (**c**) lactate production were measured in culture medium collected from WT and PHLPP knockout MEF cells. Data represent the mean ± SEM (n=3, * p<0.05 as determined by two-sample t-tests compared to the control cells). (**d**) A representative energetic map of WT, Phlpp1^–/–^ and Phlpp2^–/–^ MEF cells. The OCR and ECAR data shown in the map represent the OCR of basal respiration from the Mito stress test and the glycolysis-related ECAR from the glycolysis stress test, respectively (data represent the mean ± SEM, n=15).


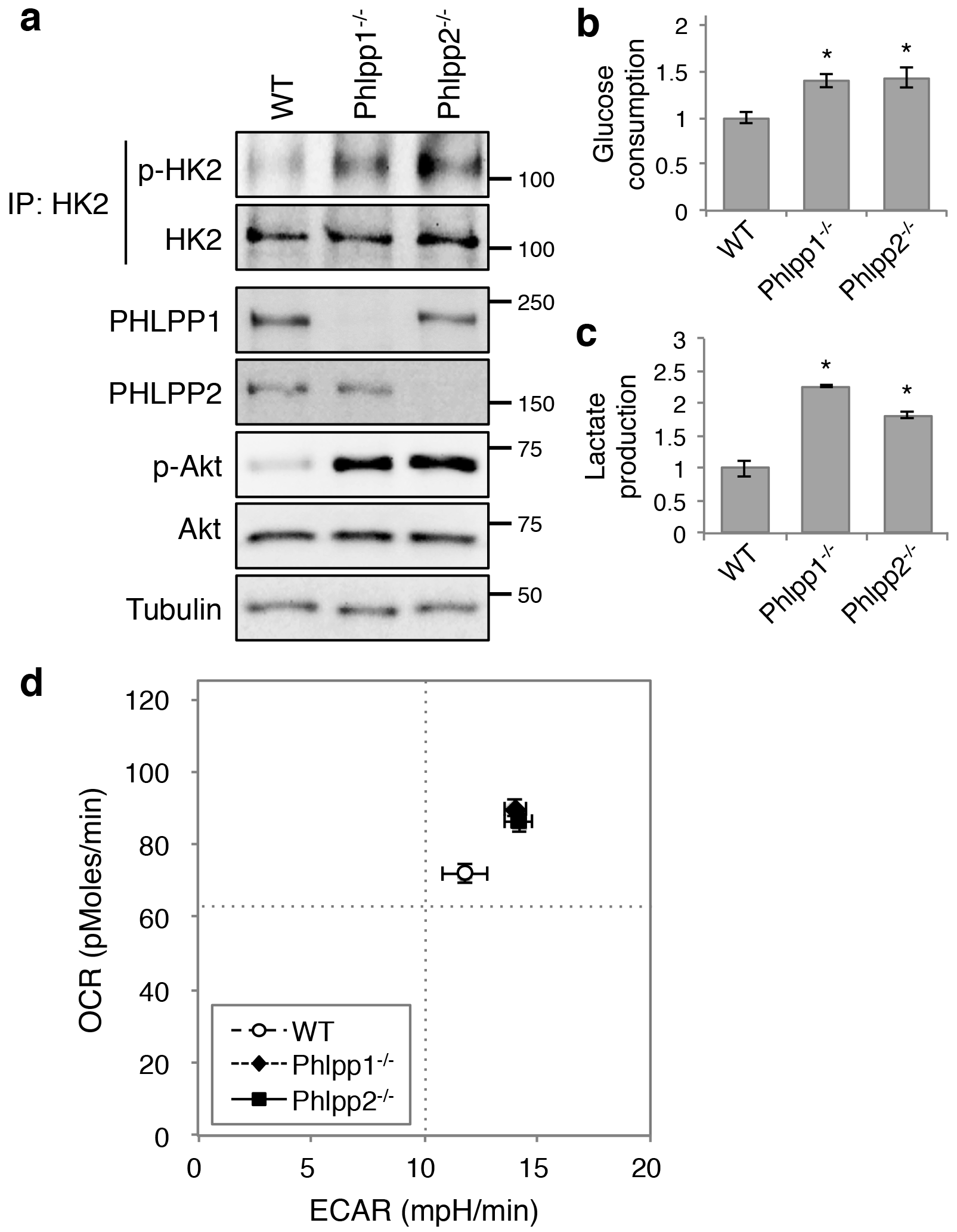


**Figure S3. Overexpression of PHLPP suppresses mitochondrial respiration in colon cancer cells.** (**a**) Representative measurements obtained from the Mito tress test performed in control (vector) and PHLPP overexpressing cells using the Seahorse XF96 Extracellular Flux analyzer. (**b**) Experiments as shown in (**a**) were quantified and the relative levels of OCR associated with basal and maximal respiration and ATP turnover were calculated based on the measurements obtained upon the addition of individual compounds. Data shown on the right represent the mean ± SEM (n=15, * p<0.05 as determined by two-sample t-tests compared to the control cells).


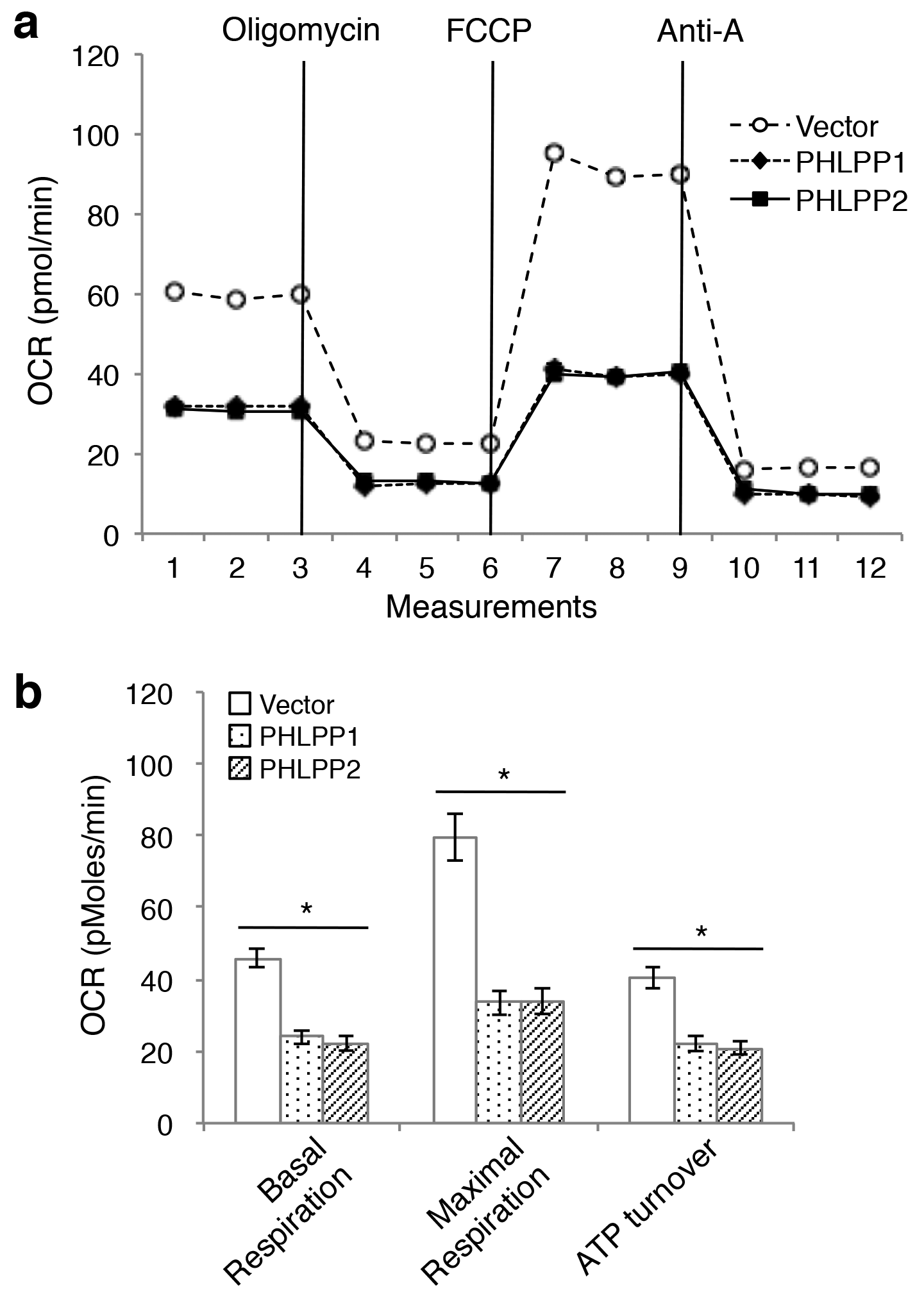


**Figure S4. Knockdown of HK2 suppresses mitochondrial respiration in colon cancer cells.** (**a**) Representative measurements obtained from the Mito stress test performed in control and HK2 knockdown cells using the Seahorse XF96 Extracellular Flux analyzer. (**b**) Experiments as shown in (**a**) were quantified and the relative levels of OCR associated with basal and maximal respiration and ATP turnover were calculated based on the measurements obtained upon the addition of individual compounds. Data represent the mean ± SEM (n=15, * p<0.05 as determined by two-sample t-tests compared to the control cells).


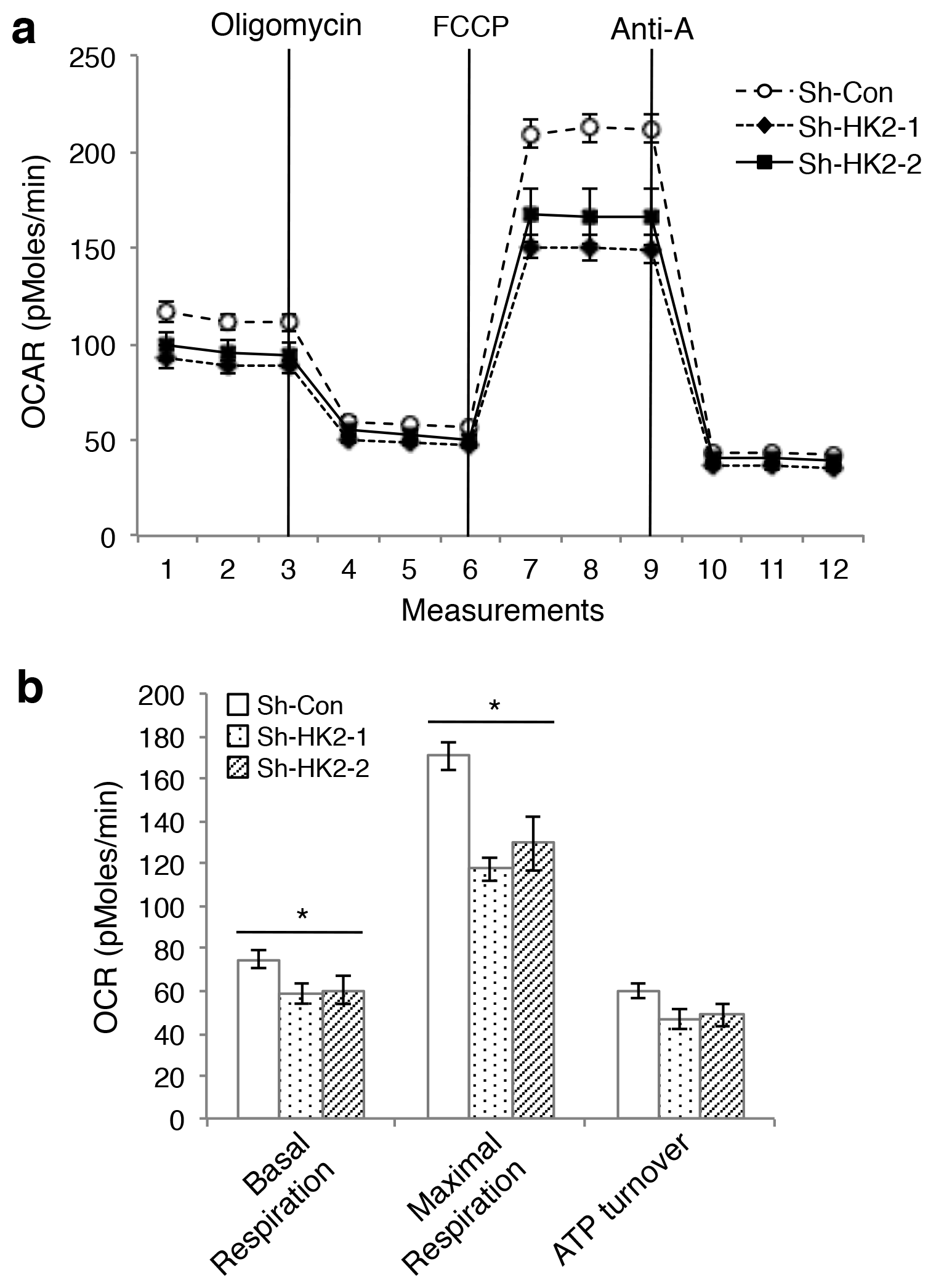

Supplement: Supplementary Information [file cddiscovery2016103-s1.doc]
